# Efficacy and Feasibility of OptraDam^®^ Plus Versus Conventional Rubber Dams in Preclinical Simulation Training: A Randomized Crossover Trial

**DOI:** 10.3390/dj13110485

**Published:** 2025-10-22

**Authors:** Fahad BaHammam, Mohammed Alsuhaibani, Faisal Almutairi, Sultan Aldakhil, Shug Albarrak, Lulwah Alreshaid, Fathima Farook

**Affiliations:** 1 College of Dentistry, King Saud bin Abdulaziz University for Health Sciences, Prince Mutib Ibn Abdullah Ibn Abdulaziz Rd, Ar Rimayah, Riyadh 14611, Saudi Arabiareshaidl@ksau-hs.edu.sa (L.A.);; 2 King Abdullah International Medical Research Centre, Ministry of the National Guard Health Affairs, Ahmad Ibn Hanbal Street, Riyadh 11481, Saudi Arabia; 3 Adams School of Dentistry, University of North Carolina at Chapel Hill, Chapel Hill, NC 27599, USA; 4 Eastman Institute for Oral Health, University of Rochester Medical Center, Rochester, NY 14620, USA

**Keywords:** cross-over studies, dental education, rubber dams, simulation training

## Abstract

**Background/Objectives:** Despite clear benefits, conventional rubber dam use remains low due to barriers that often originate during undergraduate training. To examine a potential approach to mitigating these barriers, this study evaluated the efficacy and feasibility of OptraDam^®^ Plus, a user-friendly alternative to the conventional rubber dam, in preclinical simulation training. **Methods**: In this 2 × 2 crossover trial, preclinical undergraduate students were randomly assigned to two groups to perform two types of dental isolation, conventional rubber dam and OptraDam^®^ Plus, in alternating sequences on a dental simulator. The efficacy and feasibility of both systems were evaluated based on application time, isolation quality, and students’ perceptions. **Results**: Data from 94 randomized students were collected and analyzed. Although students’ performance was suboptimal with both systems, there were significant differences in efficacy and feasibility between them. Application time was shorter with the conventional rubber dam (mean reduction 77 s; 95% CI 4–151; *p* = 0.039), whereas using OptraDam^®^ Plus was associated with 51% lower odds of achieving a higher isolation quality category (OR 0.49; *p* = 0.011). In addition, students perceived the conventional rubber dam to be superior to the OptraDam^®^ Plus in achieving higher-quality dental isolation and in their confidence in using it. **Conclusions**: OptraDam^®^ Plus cannot be considered a suitable alternative to the conventional rubber dam in preclinical simulation training due to its inferior efficacy and feasibility. The findings of this study challenge the assumption that utilization of newer marketed “user-friendly” rubber dam systems necessarily overcome the core technical barriers faced by undergraduate students.

## 1. Introduction

A rubber dam is a flexible, thin sheet made of latex or other non-latex materials [1]. The practice of dental isolation using a rubber dam was first introduced by Dr. Sanford Christie Barnum in 1864, and it continues to be a major aspect of various dental procedures such as root canal treatments and restorative work [1]. Utilization of a rubber dam during various dental procedures can offer multifaceted advantages. A rubber dam can act as a barrier that maintains moisture control and prevents salivary and microbial contamination in the working field, thereby promoting a sterile and dry environment essential for successful treatment outcomes [2]. It also serves as a protective measure for patients, significantly reducing the risk of accidental ingestion or inhalation of dental instruments or materials [3]. Additionally, it reduces atmospheric bacterial contamination, which can help minimize the risk of cross-infection in the dental clinic [4].

These benefits of isolating the operative field with a rubber dam are widely recognized by dental practitioners, yet its adoption remains low, largely due to the technical complexity and lengthy placement time [5,6]. While these challenges often arise during undergraduate training, they can go unaddressed in curricula that, despite mandating rubber dam use, fail to build adequate competence [7,8,9]. Introducing a more user-friendly isolation method early in undergraduate education could lower technical barriers, reinforce positive practice habits, and ultimately increase long-term utilization of rubber dam after graduation. Improving its long-term use has direct clinical relevance, as it could help enhance treatment outcomes and patient safety, while reducing the risk of cross-infection [1,2,3,4].

To address the need for streamlined rubber dam placement, several new generations of rubber dam systems have been introduced to the market [10]. The OptraDam^®^ (Ivoclar Vivadent, Schaan, Liechtenstein), one of these systems, was first launched in 2005 [11]. Its design integrates both the functionality of lip and cheek retractor (e.g., OptraGate^®^) with the total isolation of conventional rubber dam [11]. It features a three-dimensional anatomical design and can be used without a clamp or frame, improving time- and cost-efficiency [11]. In 2012, the OptraDam^®^ was further refined and rebranded as OptraDam^®^ Plus, with improvements suggested in comfort, ease of use, and material quality over the original OptraDam^®^ [12].

Laboratory-based and in vivo studies assessing the performance of OptraDam^®^ Plus have demonstrated that it is outperformed by the conventional rubber dam in placement time and isolation quality, especially when isolating multiple teeth [13,14]. However, in both studies, operators were likely more familiar with the conventional rubber dam, which may have biased the results in its favor. These findings may not be fully applicable to less experienced undergraduate dental students, who might face a similar learning curve with both systems.

This study, therefore, aimed to evaluate OptraDam^®^ Plus as a potential alternative to the conventional rubber dam in preclinical simulation training by comparing the efficacy and feasibility of both systems when used by preclinical undergraduate dental students. The objectives were to compare the application time, the quality of isolation (leakage prevention), and students’ preference between the two systems. The null hypotheses were that there would be no difference between the two systems in application time, isolation quality, or student preference.

## 2. Materials and Methods

### 2.1. Trial Design

This laboratory-based trial employed a randomized, controlled, two-procedure, two-period, same-session crossover design. Preclinical undergraduate dental students were randomized 1:1 to Sequence A (applying a conventional rubber dam then OptraDam^®^ Plus) or Sequence B (applying OptraDam^®^ Plus then a conventional rubber dam). Efficiency and feasibility of the two systems were assessed by application time (seconds) and isolation quality (water-retention method). Participants’ general perceptions of rubber dam isolation and their views of the two systems were collected via a paper-based questionnaire. The crossover format was used to reduce inter-participant variability and therefore decrease the number of required participants while increasing statistical power and precision.

### 2.2. Sample Size

A total sample size of 92 participants in a 2 × 2 crossover design was calculated to provide 80% power to detect significant differences in mean rubber dam application times between the two systems, using a Geisser-Greenhouse corrected F-test at a 5% significance level (α = 0.05). The sample size estimation assumed a standard deviation of 4.96 [15], across subjects at the same time point and a correlation of 0.20 between the repeated measurements. The standard deviation of the hypothesized means was 0.93. The calculation was performed using PASS software (version 15.0).

### 2.3. Participants

Inclusion criteria included undergraduate dental students enrolled in a preclinical operative dentistry course during the academic year 2024–2025 at the College of Dentistry, King Saud bin Abdulaziz University for Health Sciences (KSAU-HS), Riyadh, Saudi Arabia. Inclusion criteria also required that students had been taught the topic of dental isolation and had practiced rubber dam application in the preclinical simulation laboratory. Repeat students and those enrolled in a clinical course were excluded. Based on these criteria, a total population sampling approach was used, whereby all 106 eligible preclinical undergraduate students were invited to participate. This approach accounts for potential refusals and non-attendance, and therefore ensures that the final sample meets or exceeds the calculated required sample size.

### 2.4. Randomization

Participants were randomly and equally divided into two groups to perform the two types of dental isolation in alternating sequences to control for potential ordering effects. Participants were assigned to one of two sequences (A: conventional rubber dam then OptraDam^®^ Plus; B: OptraDam^®^ Plus then conventional rubber dam). Because the experiment was conducted in four separate sessions (each with approximately 25 participants), a biostatistician prepared four computer-generated randomization lists using Microsoft Excel. The biostatistician did not participate in recruitment, data collection, or session supervision.

Once all invited participants for a session had consented and assembled, the principal investigator matched the randomization sequence to the class list (ordered by student ID) and revealed assignments immediately before the session. Within each session, block randomization was used to ensure equal group sizes. The first block randomized 20 participants; if more than 20 attended, additional blocks of two were randomized, up to a 30th participant.

### 2.5. Interventions

All participating students were familiar with the rubber dam isolation procedure through previous didactic lectures and practical sessions. To reduce variability between participants, instructions for the placement of both rubber dam systems were provided via narrated and captioned video-recorded demonstrations that were played to all students at the beginning of each period before placing each system. These demonstrations were also uploaded online and could be accessed by the participants as much as they deemed necessary during the session. In addition, participants received written learning materials, supplemented with photographic images. All learning materials, both video and written, underwent validation by three independent faculty members from the Department of Restorative and Prosthetic Dental Sciences at the College of Dentistry, KSAU-HS. All learning materials are available upon reasonable request.

All participants performed dental isolation in a standardized manner using two rubber dam systems: a pre-stamped conventional rubber dam (Sanctuary Dental Dam Systems©, Chemor, Malaysia) and the OptraDam^®^ Plus. These procedures were conducted on a Dental Care Patient Simulator PK-1 TKUTD Head (Frasaco, Tettnang, Germany) that was mounted with Soft Gingiva Jaw Models D18-500E (Nissin Dental Products Inc., Kameoka, Japan).

In both systems, rubber dam placement occurred on the lower jaw, extending from tooth number 36 to tooth number 43. The rubber dam at tooth number 36 was secured using a Serrated Jaw Molar Winged Clamp (Ivory 13A, Kulzer, Peakhurst, Australia), and at tooth number 43 was stabilized with dental floss (Oral-B, Iowa City, IA, USA). For both systems, rubber dam placement was performed utilizing a basic set of instruments: 4 Felt/Tufts University Composite Instrument, Rubber Dam Forceps #4 (17½ cm), and Ainsworth Rubber Dam Punch (17 cm) (Hu-Friedy Group, Chicago, IL, USA). For the conventional rubber dam placement, an Adult Rubber Dam Frame RDAF6 (Hu-Friedy Group, Chicago, IL, USA) was also used. In the conventional rubber dam placement, an assembly of clamp, frame, and sheet was applied to the dental simulator using application forceps, mimicking the placement technique of the OptraDam^®^ Plus.

### 2.6. Ethical Considerations

Ethical approval was obtained from the Institutional Review Board at King Abdullah International Medical Research Center (IRB Approval Number: 0000047124). Participation was voluntary, with the option to withdraw from the study at any time. Participants received an information sheet prior to providing written informed consent. Each participant was assigned a unique, untraceable identifier to anonymize their data.

### 2.7. Measured Outcomes

There were three main outcomes measured in this study:

- The time taken for the placement of each rubber dam system by each participant, which was recorded in seconds by one of six calibrated examiners.
- The quality of isolation was assessed using the water retention ability method [13]. Following the placement of the rubber dam, one of the six calibrated examiners adjusted the lower occlusal plane to a 30-degree upward angle relative to a flat horizontal line. Then, a volume of 10 mL of water was introduced by the examiner into the isolated area with a syringe. After a period of 10 min, any residual water was aspirated back into the syringe and measured with an accuracy of ±0.5 mL. In instances where there was complete water leakage through the rubber dam, the time of occurrence was documented in seconds. The quality of isolation was evaluated based on the ordinal scale in Table 1.
- Participating students’ general perceptions and views regarding rubber dam isolation were evaluated using the first part of a paper-based questionnaire administered before the experiment, and their perceptions and views of the two tested systems were evaluated using the second part administered after the experiment (Appendix A). The questionnaire was adapted from two previously validated questionnaires [9,16]. Its face validity was evaluated by three independent faculty members in the Department of Restorative and Prosthetic Dental Sciences at the College of Dentistry, KSAU-HS.

### 2.8. Statistical Analysis

All statistical analyses were performed using RStudio (R version 4.2.2). Descriptive statistics (means, standard deviations, medians, and interquartile ranges [IQR]) were calculated for all continuous and ordinal performance variables. Responses to both parts of the questionnaire (pre- and post-experiment ordinal Likert-scale items) were summarized using medians and interquartile ranges.

To compare placement times between the two systems, a paired-samples t-test was conducted. Differences in isolation quality between the two systems were assessed using Wilcoxon signed-rank test and an ordinal logistic regression analysis. Comparative analyses of participants’ post-experiment perceptions of confidence, ease of application, and perceived isolation quality between the two systems were each assessed using Wilcoxon signed-rank tests. A significance threshold of *p* < 0.05 was applied throughout.

## 3. Results

**Figure 1:**
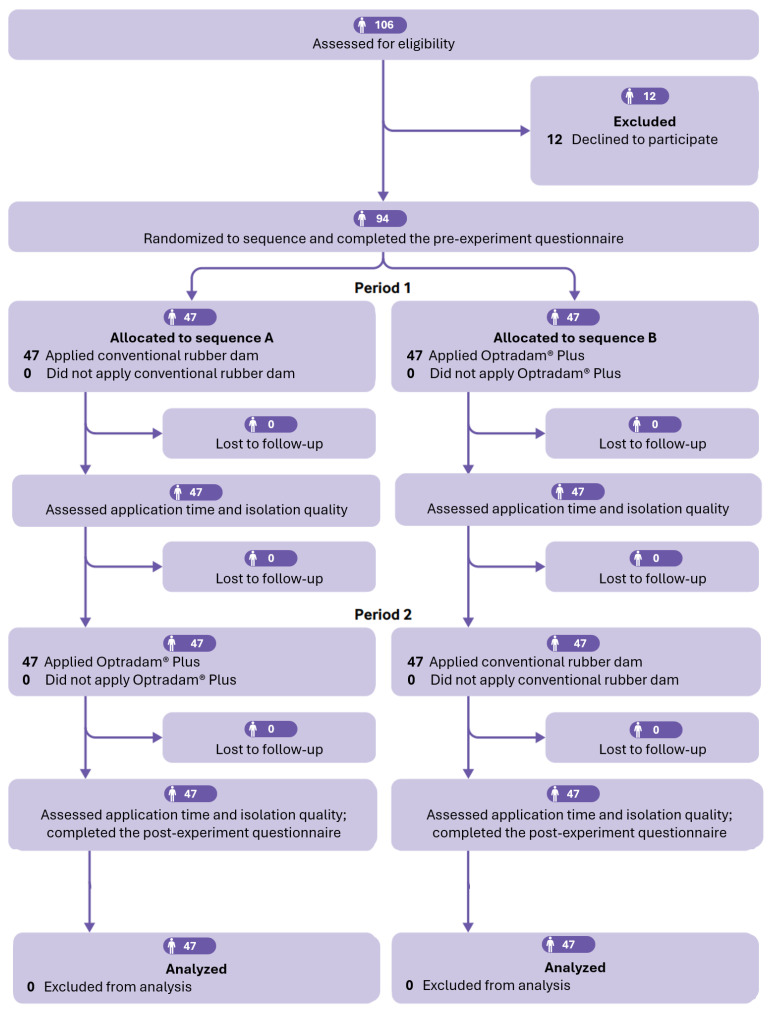
Flow diagram illustrating the study design and participant flow. Adapted from the CONSORT flow diagram [17].

Ninety-four preclinical undergraduate dental students participated in the study, of whom 50 were male. Figure 1 illustrates the study design and the participant flow throughout the trial. Data supporting the findings of this study are available in the Supplementary Materials (Dataset S1).

### 3.1. Application Time

A paired-samples *t*-test revealed a statistically significant difference in application time between the two rubber dam systems. The conventional rubber dam had a 7.8% shorter mean application time (910 ± 313 s) than the OptraDam^®^ Plus system (987 ± 348 s). This corresponds to a mean difference of 77 s (95% CI: −150.75 to −4.03 s), favoring the conventional rubber dam (t(93) = −2.09, *p* = 0.039).

### 3.2. Isolation Quality

**Figure 2:**
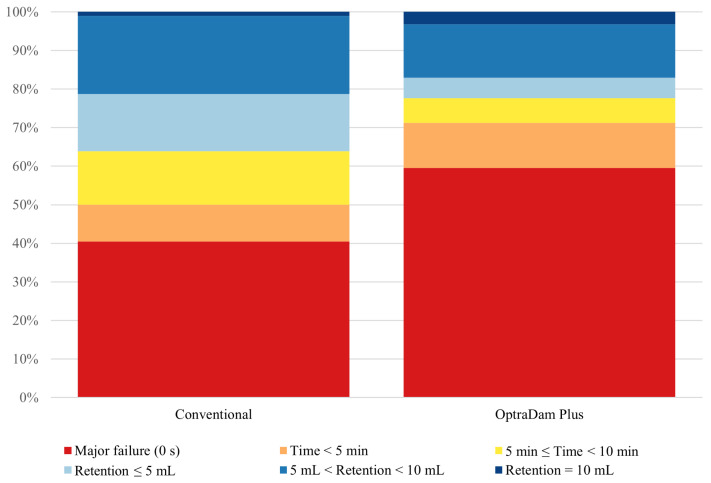
Distribution of ordinal isolation quality scores for the two rubber dam systems.

Figure 2 shows the distribution of ordinal isolation quality scores for the conventional rubber dam and OptraDam^®^ Plus. A Wilcoxon signed-rank test demonstrated significantly higher isolation quality scores with the conventional rubber dam (median = 1.5) compared to the OptraDam^®^ Plus (median = 0; V = 1204.5, *p* = 0.015). In addition, an ordinal logistic regression revealed that using OptraDam^®^ Plus was associated with 51% lower odds of achieving a higher isolation quality score compared to the conventional rubber dam (OR = 0.49, *p* = 0.011), with a regression coefficient of −0.714 (SE = 0.280; t = −2.555).

### 3.3. Pre-Experiment Perceptions

**Figure 3:**
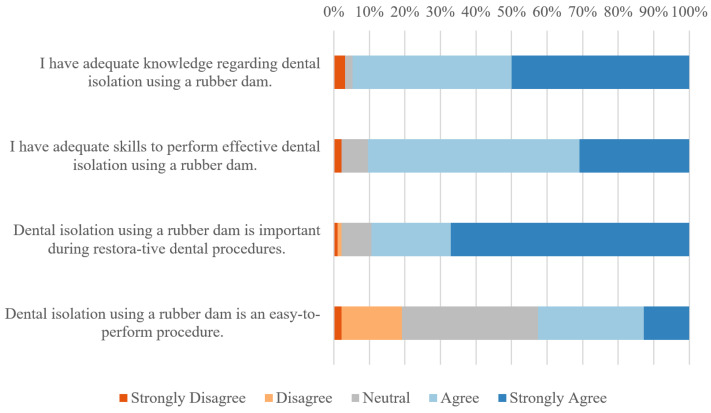
Pre-experiment perceptions of rubber dam isolation.

Participants mostly reported favorable baseline perceptions of rubber dam isolation (Figure 3 and Table 2). Although they rated their knowledge, their skills, and the perceived importance highly, the perceived ease of application was notably lower.

### 3.4. Post-Experiment Perceptions

**Figure 4:**
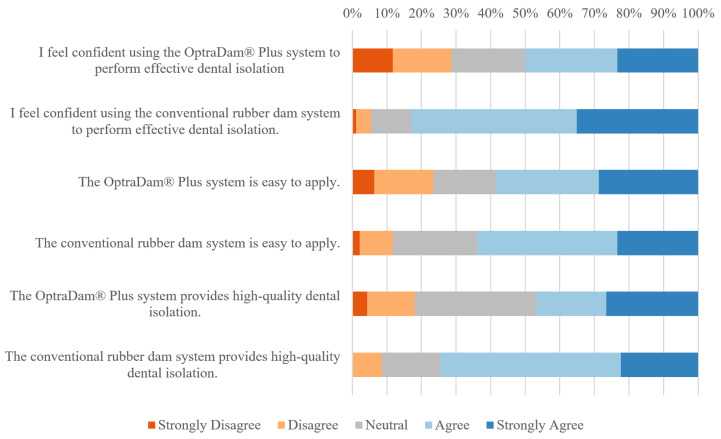
Post-experiment perceptions of the two isolation systems.

After the experiment, participants generally favored the conventional rubber dam over OptraDam^®^ Plus (Figure 4 and Table 3). Statistically significant findings from Wilcoxon signed-rank tests indicated that participants perceived the conventional rubber dam to be superior to the OptraDam^®^ Plus in achieving higher-quality dental isolation and in their confidence to do so. However, there was no statistically significant difference in participants’ perceptions between the two systems regarding ease of use.

## 4. Discussion

This laboratory-based randomized crossover trial demonstrated that OptraDam^®^ Plus is an inferior alternative to the conventional rubber dam for enhancing the efficacy and feasibility of rubber dam isolation during preclinical simulation training. Statistically significant results showed that the conventional rubber dam required less application time and produced superior isolation quality when used by preclinical undergraduate students. These students also perceived the conventional rubber dam as superior for achieving high-quality isolation and reported greater confidence in using it. These findings are consistent with previous laboratory-based and in vivo studies, in which more experienced operators required less application time, achieved superior isolation quality, and reported greater acceptance of the conventional rubber dam compared with OptraDam^®^ Plus [13,14,18].

While the findings of this study clearly indicated that the conventional rubber dam outperforms OptraDam^®^ Plus, it is possible that the observed superiority is due to factors other than the inherent properties of the two systems. For instance, the participating students were slightly more familiar with the conventional rubber dam, which is the only system taught and used at the college. In contrast, all participating students applied OptraDam^®^ Plus for the first time during this trial. This limitation could have been mitigated by recruiting the students at a stage when they had no prior experience in rubber dam isolation. However, this approach was not adopted because the students at that stage were anticipated to lack the basic skills, potentially creating a floor effect that would prevent any differences between the two systems from being detected [19]. Another approach, which was not implemented due to feasibility constraints, would have been to require the students to apply both rubber dam systems multiple times during the trial to narrow the learning-curve gap between them. However, the effectiveness of this approach is questionable, as Kapitán et al. (2014) demonstrated only minimal improvement in operators’ performance with OptraDam^®^ Plus even after 60 applications [13].

Another possible factor that may have confounded the results of this trial is the use of dental simulators and jaw models, which do not perfectly match human anatomy. OptraDam^®^ Plus features a three-dimensional anatomical design suited to the human oral cavity [11]; therefore, the mismatch between OptraDam^®^ Plus design and the simulators and models may have contributed to its inferior performance in this trial. Nonetheless, the effect of this mismatch is not supported by clinical studies evaluating both systems, which generally indicate a superior performance for the conventional rubber dam [14,15,18]. An additional contributing factor that may have increased the application time of OptraDam^®^ Plus was the use of a clamp, despite it being marketed for use without one [11]. The use of a clamp with OptraDam^®^ Plus in this trial was based on the findings from previous studies and customer reports, which indicated that achieving adequate isolation with OptraDam^®^ Plus in posterior teeth was challenging without the use of a clamp [14,20].

In this trial, the application time for the conventional rubber dam was 7.8% shorter than that for OptraDam^®^ Plus. The magnitude of this reduction was significantly smaller than that reported in other studies when more experienced operators tested both systems [13,14]. For example, Kapitán et al. (2014) reported 15% shorter time in a laboratory-based study, and Kapitán et al. (2015) reported a 26% shorter time in an in vivo study [13,14]. These differences in magnitude between the current study and previous ones may be attributable to operators’ greater familiarity with the conventional rubber dam. This suggests that among less experienced operators, the feasibility differences between the two systems might be less pronounced.

Similarly, the conventional rubber dam, in this trial, outperformed OptraDam^®^ Plus in isolation quality, as OptraDam^®^ Plus was associated with a 51% lower odds of achieving a higher isolation quality score compared to the conventional rubber dam. This magnitude of difference is comparable to other studies that were performed by more experienced operators [13,14]. For example, Kapitán et al. (2014) reported that conventional rubber dam retained approximately twice the water volume of OptraDam^®^ Plus when evaluated using the same method as in this study [13]. This may suggest that operator experience is similarly associated with the efficiency of both systems.

A notable finding of this study was the suboptimal performance of the participating students in applying rubber dams. This may represent the first objective empirical evidence that validates numerous previous reports of students’ negative perceptions regarding their technical competence [21,22]. The mean application time for both systems was approximately 16 min, about three times as long as that reported for senior dental students and eight times as long as that reported for senior dentists [13,22]. In addition, although the participating students achieved better isolation quality with the conventional rubber dam, they did not achieve an acceptable level of isolation with either system. Of 188 applications across both systems, a perfect isolation score was attained on only four occasions. Conversely, the majority experienced major failures that would necessitate repetition of the isolation procedure (approximately 40% of conventional rubber dam applications and 60% of OptraDam^®^ Plus applications). This finding contrasts with a previous laboratory-based study conducted with more experienced operators, which reported a significantly lower proportion of major failures [13]. This suboptimal performance likely reflects the participating students’ limited clinical experience at the time of the trial. Therefore, it is reasonable to anticipate further improvement as they advance through their undergraduate years [23].

Although the participating students did not perceive rubber dam isolation as an easy task, they had favorable baseline perceptions that their skills and knowledge were adequate to perform it effectively. This perceived adequacy, especially within the skills domain, contrasts with their actual performance and is not in line with findings from several questionnaire-based studies conducted worldwide that have explored undergraduate dental students’ perceptions of rubber dam isolation [21,22,24]. The contrasting perceptions may reflect the Dunning–Kruger effect, as previous studies examined more senior students who were probably more knowledgeable and skilled and therefore less prone to overestimate their abilities [25]. Accordingly, objective feedback introduced early in the undergraduate years to calibrate self-assessment may mitigate this effect, thereby supporting more effective skill development throughout the undergraduate program [26]. Social desirability bias is another possible explanation of the skills overestimation in this study, given that some students might want to please certain research team members who were directly involved in teaching the topic of dental and rubber dam isolation [27].

While students perceived both systems to be equal in terms of ease of use, they viewed the conventional rubber dam to be superior to OptraDam^®^ Plus in achieving higher-quality dental isolation and reported greater confidence in achieving high-quality isolation with the conventional rubber dam. These differences in perceptions of isolation quality between the systems are consistent with the participants’ actual performance. They are also in line with a previous clinical study that surveyed dental practitioners about the two systems, which revealed that while practitioners did not perceive any differences in terms of ease of use, they perceived isolation quality to be superior when using the conventional rubber dam [18]. This may explain why consistent reports from dental practitioners favor the conventional rubber dam over OptraDam^®^ Plus [18,28].

To achieve robust findings in this trial, several steps were undertaken to ensure the strength of the methods applied. The sample size was calculated in advance to provide adequate power to detect statistically significant differences in the primary outcome (i.e., application time in seconds). In addition, this trial employed a randomized two-period crossover design, which controls for inter-participant variability and increases precision by using each participant as their own control. Participants were randomized to two sequences to balance learning effects. This was done because it was suspected that participants’ performance would improve in the second period. Furthermore, all participants applied the two systems using the same, standardized technique; materials and instruments were uniform. Moreover, objective measures and a face-validated questionnaire were used to collect the data in this study.

Due to feasibility constraints, this study assessed only one multi-tooth isolation scenario (teeth 36–43, crossing the mandibular midline). This limitation is noteworthy because Kapitán et al. (2014) reported that OptraDam^®^ Plus can outperform the conventional rubber dam in single-tooth isolation [13]. Accordingly, OptraDam^®^ Plus may be superior to the conventional rubber dam in some isolation contexts beyond the one tested in this study. Thus, it is crucial to evaluate both systems across a broader range of isolation scenarios, including single-tooth cases, when they are performed by students during preclinical simulation training. Another limitation in this study is that participants were more familiar with the conventional rubber dam, which may have biased the results in its favor. In addition, as this trial was conducted in a laboratory setting, its findings may not be fully generalizable to clinical settings. Moreover, it was not possible to blind the participants or the evaluators to the two systems due to their distinct designs, which may introduce performance and observer biases.

Future research should address the ongoing challenges that dental students continue to face with rubber dam application. This can involve investigating the effectiveness of novel approaches in teaching the topic of rubber dam isolation. For instance, promising but inconclusive evidence has been published on the potential benefits of virtual-reality and video-based modalities [29,30]. Understanding the underlying causes of these challenges from the students’ perspective through qualitative research may further help address these challenges.

## 5. Conclusions

Rubber dam application remains a challenging task for many preclinical undergraduate students, as demonstrated in this study by the prolonged application times and suboptimal isolation quality with both OptraDam^®^ Plus and the conventional rubber dam. OptraDam^®^ Plus cannot be considered a suitable alternative to the conventional rubber dam due to its inferior efficacy and feasibility in preclinical simulation training. This challenges the assumption that newer, marketed “user-friendly” systems necessarily overcome the fundamental technical barriers of rubber dam application for undergraduate students. Future research should address these challenges by understanding their underlying causes and developing and evaluating novel educational approaches.

## Figures and Tables

**Table 1:** Ordinal scale for isolation quality evaluation.

Score	Definition
0	Major failure of the rubber dam, resulting in immediate full water leakage.
1	Complete water leakage occurs within 5 min.
2	Complete water leakage occurs between 5 and 10 min.
3	After 10 min, retains ≤5 mL of the 10 mL water volume.
4	After 10 min, retains >5 mL but <10 mL of the water volume
5	After 10 min, retains all 10 mL of the water volume.

**Table 2:** Summary statistics of participants’ pre-experiment perceptions.

No.	Items	Median (IQR) ^1^
1	I have adequate knowledge regarding dental isolation using a rubber dam.	4.5 (4.0–5.0)
2	I have adequate skills to perform effective dental isolation using a rubber dam.	4.0 (4.0–5.0)
3	Dental isolation using a rubber dam is important during restorative dental procedures.	5.0 (4.0–5.0)
4	Dental isolation using a rubber dam is an easy-to-perform procedure.	3.0 (3.0–4.0)

^1^ A five-point scale was used, and a higher score indicated greater agreement with the statement.

**Table 3:** Summary statistics of participants’ post-experiment perceptions and Wilcoxon comparisons.

Variable	OptraDam^®^ Plus Median (IQR)	Conventional Median (IQR)	Wilcoxon V	*p*-Value
I feel confident using this system.	3.5 (2.0–4.00)	4.0 (4.0–5.0)	385	<0.001 *
This system is easy to apply.	4.0 (3.0–5.0)	4.0 (3.0–4.0)	1269	0.306
This system provides high-quality dental isolation	3.0 (3.0–5.0)	4.0 (3.5–4.0)	661	0.009 *

A five-point scale was used, and a higher score indicated greater agreement with the statement. * Indicates statistical significance at *p* < 0.05.

## Data Availability

Data is contained within the Supplementary Material.

## Supplementary Materials

The following supporting information can be downloaded at https://www.mdpi.com/article/10.3390/dj13110485/s1: Dataset S1.

## Abbreviations

IRB	Institutional Review Board
IQRs	Interquartile ranges
KAIMRC	King Abdullah International Medical Research Center
KSAU-HS	King Saud bin Abdulaziz University for Health Sciences

## Appendix A

Pre-experiment questionnaire
I have adequate knowledge regarding dental isolation using a rubber dam.
  1. Strongly Disagree
  2. Disagree
  3. Neutral
  4. Agree
  5. Strongly Agree
I have adequate skills to perform effective dental isolation using a rubber dam.
  1. Strongly Disagree
  2. Disagree
  3. Neutral
  4. Agree
  5. Strongly Agree
Dental isolation using a rubber dam is important during restorative dental procedures.
  1. Strongly Disagree
  2. Disagree
  3. Neutral
  4. Agree
  5. Strongly Agree
Dental isolation using a rubber dam is an easy-to-perform procedure.
  1. Strongly Disagree
  2. Disagree
  3. Neutral
  4. Agree
  5. Strongly Agree
Post-experiment questionnaire
The OptraDam^®^ Plus system is easy to apply.
  1. Strongly Disagree
  2. Disagree
  3. Neutral
  4. Agree
  5. Strongly Agree
The conventional rubber dam system is easy to apply.
  1. Strongly Disagree
  2. Disagree
  3. Neutral
  4. Agree
  5. Strongly Agree
I feel confident using the OptraDam^®^ Plus system to perform effective dental isolation.
  1. Strongly Disagree
  2. Disagree
  3. Neutral
  4. Agree
  5. Strongly Agree
I feel confident using the conventional rubber dam system to perform effective dental isolation.
  1. Strongly Disagree
  2. Disagree
  3. Neutral
  4. Agree
  5. Strongly Agree
The OptraDam^®^ Plus system provides high-quality dental isolation.
  1. Strongly Disagree
  2. Disagree
  3. Neutral
  4. Agree
  5. Strongly Agree
The conventional rubber dam system provides high-quality dental isolation.
  1. Strongly Disagree
  2. Disagree
  3. Neutral
  4. Agree
  5. Strongly Agree
